# Motion-Defined Form Perception in Deprivation Amblyopia

**DOI:** 10.1167/iovs.65.4.13

**Published:** 2024-04-04

**Authors:** Deborah E. Giaschi, Akosua K. Asare, Reed M. Jost, Krista R. Kelly, Eileen E. Birch

**Affiliations:** 1 Department of Ophthalmology and Visual Sciences, University of British Columbia, Vancouver, British Columbia, Canada; 2 Pediatric Vision Laboratory, Retina Foundation of the Southwest, Dallas, Texas, United States; 3 School of Optometry and Vision Science, University of Waterloo, Waterloo, Ontario, Canada

**Keywords:** amblyopia, motion perception, binocular vision, pediatric cataract

## Abstract

**Purpose:**

The purpose of this study was to assess motion-defined form perception, including the association with clinical and sensory factors that may drive performance, in each eye of children with deprivation amblyopia due to unilateral cataract.

**Methods:**

Coherence thresholds for orientation discrimination of motion-defined form were measured using a staircase procedure in 30 children with deprivation amblyopia and 59 age-matched controls. Visual acuity, stereoacuity, fusion, and interocular suppression were also measured. Fixation stability and fellow-eye global motion thresholds were measured in a subset of children.

**Results:**

Motion-defined form coherence thresholds were elevated in 90% of children with deprivation amblyopia when viewing with the amblyopic eye and in 40% when viewing with the fellow eye. The deficit was similar in children with a cataract that had been visually significant at birth (congenital) and in children for whom the cataract appeared later in infancy or childhood (developmental). Poorer motion-defined form perception in amblyopic eyes was associated with poorer visual acuity, poorer binocular function, greater interocular suppression, and the presence of nystagmus. Fellow-eye deficits were not associated with any of these factors, but a temporo-nasal asymmetry for global motion perception in favor of nasalward motion suggested a general disruption in motion perception.

**Conclusions:**

Deficits in motion-defined form perception are common in children with deprivation amblyopia and may reflect a problem in motion processing that relies on binocular mechanisms.

Disrupted visual experience in early childhood hinders visual development and can lead to amblyopia. Unilateral anisometropia and/or strabismus are the most common causes of amblyopia, and the defining deficit is reduced visual acuity in the affected eye that cannot be immediately improved with refractive correction. It is now well established that deficits in other aspects of vision, such as motion perception, contrast sensitivity, spatial integration, and reading, as well as ocular motor and visuomotor deficits, are common in anisometropic and strabismic amblyopia.^1^^–^^4^ Many of these deficits are also found in the clinically unaffected fellow eye that has normal visual acuity.^5^^,^^6^ The involvement of both eyes in these additional deficits implicates disruption of binocular mechanisms and supports the growing evidence that amblyopia should be treated as a binocular rather than a monocular disorder.^7^^,^^8^ The pattern of visual deficits in amblyopia has been shown to depend on the presence or absence of residual binocular function.^9^

Amblyopia is less commonly caused by form deprivation, and this is most often due to unilateral cataract. This type of deprivation usually results in poorer visual acuity outcomes than anisometropia or strabismus,^10^^,^^11^ and nil or severely reduced stereoacuity.^12^ Although deprivation amblyopia is a more common animal model,^13^^–^^15^ it has been less studied in humans than anisometropic or strabismic amblyopia, and it remains unclear if the underlying neural mechanisms are the same or not. The purpose of this study was to assess motion-defined form perception in each eye of children with deprivation amblyopia due to unilateral cataract. Because this aspect of vision appears to be very sensitive to early disruption by binocularly discordant visual experience,^16^^–^^19^ as reviewed below, it may provide important clues to commonalities or differences in the neural mechanisms underlying different etiological subtypes of amblyopia.

Motion-defined form perception is the ability to identify or discriminate between two-dimensional shapes defined by relative motion rather than by luminance contrast. It is often impaired in individuals with anisometropic and/or strabismic amblyopia with viewing through either the amblyopic or the fellow eye,^16^^,^^17^^,^^20^ and particularly for slow speeds of motion.^21^ Motion-defined form perception is also impaired in people with disrupted binocular vision due to unilateral enucleation.^18^^,^^19^ This may be considered to be a more complete form of monocular deprivation than that produced by a cataract, but enucleated individuals are also lacking the interocular suppression that characterizes all types of unilateral amblyopia.^22^^–^^24^

Motion-defined form is a type of second-order form stimulus^25^ that is often created using different directions of global dot motion inside and outside a stationary shape. A performance threshold is obtained by determining the smallest proportion of dots moving in a coherent direction that is required to see the shape. Thus, a deficit in performance on this task could represent a problem with complex form perception or with the integration of local motion signals to create a global motion percept. Deficits in second-order form perception based on texture contrast have been reported in the amblyopic eye in unilateral deprivation amblyopia.^26^ Deficits in global motion perception have been reported in both amblyopic and fellow eyes in unilateral deprivation amblyopia.^27^ These deficits are similar to those reported for anisometropic and strabismic amblyopia, but are relatively small compared to the deficits observed in bilateral deprivation amblyopia.^23^^,^^28^^–^^30^ In contrast, unilateral enucleation spares perception of second-order form defined by texture contrast, but disrupts global motion perception such that coherence thresholds are higher for temporalward motion and lower for nasalward motion.^18^ A preliminary report suggests a similar nasal/temporal asymmetry in global motion perception may be present with fellow-eye viewing in deprivation amblyopia.^31^ The interaction of motion and form cues has also been studied using dynamic glass patterns. Implied motion thresholds for local and global processing of these patterns were normal in anisometropic amblyopia and slightly elevated in strabismic amblyopia.^32^ Thus, the motion and form deficits in amblyopia may be stimulus specific. To our knowledge, dynamic glass patterns have not been studied in deprivation amblyopia or unilateral enucleation.

Our primary aim was to assess motion-defined form perception in the amblyopic and fellow eyes of children with deprivation amblyopia and age-matched controls, using stimuli that have previously revealed large deficits in anisometropic and strabismic amblyopia.^21^ In secondary analyses, we investigated clinical and sensory factors that may drive performance in the motion-defined form task, including age at which a visually significant cataract was first present, binocular function, interocular suppression, fixation stability, and global motion perception.

## Methods

### Participants

Eighty-nine participants aged 4 to 15 years were involved in this study: 30 children with deprivation amblyopia (mean age = 8.9 years, SD = 3.2) and 59 age-matched controls (mean age = 9.2 years, SD = 2.5) with healthy vision. For the amblyopia group, children were eligible to participate based on a diagnosis of deprivation amblyopia by a referring ophthalmologist, and a history of cataract surgery, optical correction, and occlusion therapy. Children were excluded if they had: gestational age <32 weeks, developmental delay, learning difficulty, or any other co-existing ocular or systemic disease. The amblyopia group consisted of 11 children with congenital cataracts (diagnosis of a visually significant cataract by 1.5 months) and 19 with developmental cataracts (diagnosis at ≥7 months) who had successfully undergone cataract surgery. Eligible controls had monocular visual acuity of 0.1 logMAR or better and Randot Preschool Stereoacuity^33^ of 2.0 log arcsec or better (in habitual refractive correction, if needed), no history of surgery, and no ocular or systemic disease.

This study was conducted in accordance with the tenets of the Declaration of Helsinki and complied with the Health Insurance Portability and Accountability Act. The Institutional Review Board at the University of Texas Southwestern Medical Center approved all procedures of the study. Written informed consent was obtained from each participant's parent and, for children aged 10 years or older, the child's assent was also obtained after the study had been explained.

### Visual Assessment

All 30 participants in the deprivation amblyopia group underwent a comprehensive eye examination by a pediatric ophthalmologist. Clinical data on etiology, age at cataract diagnosis, and surgery date were obtained from medical records. During the study visit, best-corrected visual acuity was assessed monocularly, using the Electronic Early Treatment Diabetic Retinopathy Study E-ETDRS^34^ (children ≥7 years) or Amblyopia Treatment Study (ATS)-HOTV^35^ (children <7 years) protocol. Binocular function scores were derived from results of random dot stereoacuity tests (Randot Preschool Stereoacuity Test [2.9–1.6 log arcsec; 800–40 arcsec, and Stereo Butterfly Test [3.3–3.1 log arcsec; 2000–1150 arcsec]; Stereo Optical Co., Inc., Chicago, IL, USA) and the Worth-4-dot test at 33 cm.^36^ Binocular function score was recorded as the stereoacuity in log arcsec for the smallest disparity Randot target that was correctly identified. If stereoacuity was nil, a binocular function score of 4.0 was assigned if fusion was present for the Worth 4-Dot Test, and 5.0 was assigned if suppression was present for the Worth 4-Dot Test.

Extent of interocular suppression was assessed with the Worth-4-Dot test at seven viewing distances (3 m to 0.16 m). The maximum distance at which a participant could see all four dots (fusion) was noted and provided an estimate of suppression scotoma size in log degrees.^37^ If no fusion was present at the closest distance (0.16 m), a nominal value of 1.35 log degrees was assigned.

Depth of suppression was measured using a computer-generated dichoptic eye chart.^38^^,^^39^ Each line of the eye chart comprised five letters, and each eye saw a different eye chart. At each position, the identity of the letter and its contrast varied independently for each eye, but the sum of the left and right eye contrasts was always 100%. Participants were instructed to name the letters from left to right. An adaptive QUEST algorithm was used to obtain a balance point, that could range from 0.5 (equally balanced) to 1.0 (complete suppression), based on the contrast at which 50% of the letters seen by the amblyopic eye were reported (right eye in the controls). This was converted to a contrast balance index (CBI) by calculating the ratio of balance point to (1-balance point). A CBI of 1.0 indicates an equal balance between the 2 eyes and higher numbers suggest increasing suppression. A score of 11 was assigned whenever the CBI exceeded 10. This was done to avoid exaggerating the depth of suppression because values above 10 rose exponentially with only small changes in the contrast balance point.

Fixation stability of each eye was assessed in a subset of children in the amblyopia group (18 amblyopic eyes and 21 fellow eyes) under monocular viewing of a 0.3 degrees diameter dot for 20 seconds using a 500 Hz remote video eye tracker (EyeLink 1000; SR Research). Qualitatively, each eye movement record was categorized for the presence or absence of fusion maldevelopment nystagmus (FMNS) and infantile nystagmus.^40^ The bivariate contour ellipse area (BCEA; log degree^2^) was used as a measure of fixation stability for each eye.^41^^,^^42^

### Motion-Defined Form

The motion-defined form stimulus^20^^,^^21^^,^^43^ comprised a random array of white dots (1.7 arcmin^2^; 150 cd/m^2^) on a black background (11.5 × 6.6 degrees, 1.0 cd/m^2^) with a dot density of 8% (170 dots/degrees^2^) and at a dot speed of 0.08 degrees/second. This is the speed that is most sensitive to disruption by strabismic and/or anisometropic amblyopia.^21^ Dots inside a rectangular region moved vertically up or down on a particular trial, and dots outside this region moved in the opposite direction at the same speed. This relative motion created a 1 × 2 degrees stationary rectangle in the center of the screen that was oriented vertically or horizontally on each trial. The task was to indicate the orientation of this motion-defined rectangle. These stimuli are illustrated in a video in the supplementary material. The proportion of coherently moving dots, both inside and outside the rectangle, was progressively decreased from 1.0 using a 2-down 1-up staircase. The initial step size of 0.2 was halved after 2 reversals and halved again after 4 reversals. The staircase terminated after 50 trials or 8 reversals, whichever came first. The coherence threshold was computed as the arithmetic mean of the last six reversals.

To ensure that each child understood the task, practice trials were conducted binocularly before the monocular staircases. In the context of spotting enemy spaceships in a Star Wars–like game, the child was taught to indicate the orientation of the spaceship (motion-defined rectangle) as tall or long by pointing to the rectangle with the same orientation on a matching card. If the child was able to do this successfully, the concept of reduced motion coherence was then introduced as “disruption of radar that causes randomly moving stars” and the child was asked to match the orientation of the enemy spaceship even though the radar was disrupted. If the child was able to perform this task correctly for two trials (one in each orientation), the monocular testing was initiated starting with the fellow eye; otherwise, the test was aborted.

### Global Motion

When time and cooperation permitted, global motion perception^32^^,^^44^ also was assessed in the right eye of controls (*n* = 17) and the fellow eye of children in the deprivation amblyopia group (*n* = 13) to explore whether there was a direct link between performance on global motion and motion-defined form tasks. Viewing random dot kinematograms (6 degrees/second, 100 dots, 1.27 dot/degrees^2^, 0.1 arcmin dot spacing, 17 ms frame duration), the child was asked to indicate whether the coherent subset of dots were moving left or right by pointing in the direction of motion or saying that the dots were swimming toward a picture of Squirt (left) or a picture of Nemo (right). Nasalward and temporalward motion coherence thresholds were measured using 2 interleaved, 2-down 1-up adaptive staircases, each starting at a coherence of 1.0. Step size was relative to the progress of the staircase; for the first 2 reversals, the step size was 50% of the current coherence level and, for the last 6 reversals, the step size was 25% of the current coherence level. The staircase was aborted if 10 responses were correct at the minimum coherence of 0.2 or if 10 were incorrect at the maximum coherence of 1.0. Temporalward and nasalward coherence thresholds were determined from the mean of the last six reversals, and the temporalward to nasalward coherence ratio was computed (T/N ratio).

### Data Analysis

Mean differences in motion-defined form thresholds in amblyopic and fellow eyes versus control eyes were assessed using separate independent sample t-tests. In addition, motion-defined form thresholds from controls were used to determine a 95% upper limit of normal performance, defined as the mean coherence threshold +1.645 SD. The number of children exceeding this limit in each eye was determined. The mean and standard deviation of the control group were also used to convert the motion-defined form threshold of each child in the amblyopia group into a z-score. The z-scores for each eye of the 11 children with a history of congenital unilateral cataract and the 19 children with developmental cataracts were compared using a 2-way mixed-design ANOVA (2 groups × 2 eyes).

The effect of sensory factors was assessed with *t*-tests to compare motion-defined form thresholds in children with mild/moderate versus severe amblyopia, and in children with and without nystagmus. For fixation instability and global motion coherence threshold ratios, upper limits on normal performance (+1.645 SD from control mean) were calculated, and the number of children exceeding this limit was determined. Associations between motion-defined form thresholds and sensory factors were assessed with parametric (visual acuity, fixation stability, and global motion) or nonparametric (binocular function and interocular suppression) correlation analyses, as appropriate for the type of data scale. Factors with at least moderate correlations were then examined together using multiple regression to determine which were significant predictors of motion-defined form coherence thresholds.

## Results

Participants’ clinical and visual assessment data are listed in the Table.

**Table.** Clinical and Visual Assessment Data

	Test Age, Y	Visually Significant Cataract Present, m	Age at Surgery, m	Amblyopic Eye VA, logMAR	Fellow Eye VA, logMAR	Binocular Function Score	Contrast Balance Index
Dense congenital							
1	6.3	0.0	0.3	0.50	−0.10	5.00	11.00
2	6.1	0.0	1.8	0.30	0.20	5.00	3.55
3	6.1	0.0	1.8	0.50	0.10	5.00	11.00
4	7.0	0.0	1.0	0.20	−0.10	4.00	1.86
5	7.7	0.0	1.8	0.40	0.00	4.00	11.00
6	6.3	0.2	1.1	1.00	0.00	5.00	11.00
7	7.0	0.5	0.7	0.80	0.10	5.00	11.00
8	13.3	0.6	0.9	2.00	0.00	5.00	11.00
9	13.5	0.6	0.8	1.70	0.00	5.00	11.00
10	6.5	0.7	1.1	0.20	−0.10	5.00	3.76
11	13.6	1.4	1.4	0.60	0.10	5.00	2.85
Developmental							
12	13.6	7.4	9.1	1.20	−0.10	4.00	1.44
13	5.6	8.0	8.7	0.40	0.00	5.00	6.69
14	4.7	8.4	8.7	0.80	−0.10	5.00	11.00
15	14.6	10.1	11.8	1.20	0.00	5.00	11.00
16	5.6	11.7	12.3	0.50	0.10	5.00	11.00
17	6.2	12.4	12.7	1.70	−0.10	5.00	11.00
18	13.5	12.5	17.1	0.60	−0.10	3.10	11.00
19	5.9	15.4	15.5	0.10	0.00	4.00	6.69
20	8.9	16.0	17.8	1.20	0.00	5.00	11.00
21	11.5	16.1	16.5	1.00	0.10	4.00	11.00
22	8.3	17.1	17.6	0.60	0.10	5.00	4.00
23	7.4	17.5	19.4	0.10	−0.10	2.30	4.26
24	6.5	19.8	20.9	1.20	0.00	5.00	11.00
25	9.5	33.6	35.3	0.40	−0.10	2.30	11.00
26	11.7	36.7	36.8	0.40	0.20	2.80	1.78
27	13.3	51.4	52.0	1.50	0.00	5.00	11.00
28	6.5	60.0	60.2	0.20	0.00	4.00	9.00
29	9.5	71.3	74.5	0.60	0.00	3.10	7.33
30	12.3	104.3	105.3	0.80	−0.10	3.10	7.33
Overall							
N	30	30	30	30	30	30	30
Mean	8.9	17.8	18.8	0.76	0.00	4.4	8.25
SD	3.2	24.7	24.8	0.51	0.09	0.9	3.57
Congenital							
N	11	11	11	11	11	11	11
Mean	8.5	0.4	1.2	0.75	0.02	4.8	8.06
SD	3.2	0.4	0.5	0.60	0.10	0.40	4.06
Developmental							
N	19	19	19	19	19	19	19
Mean	9.2	27.9	29.1	0.76	−0.01	4.09	8.34
SD	3.2	26.2	26.3	0.47	0.09	1.01	3.37
Control							
N	59	NA	NA	NA	59	59	55
Mean	9.2	NA	NA	NA	OD: −0.05	1.6	1.06
SD	2.5	NA	NA	NA	0.07	0.2	0.28

**Figure 1.**
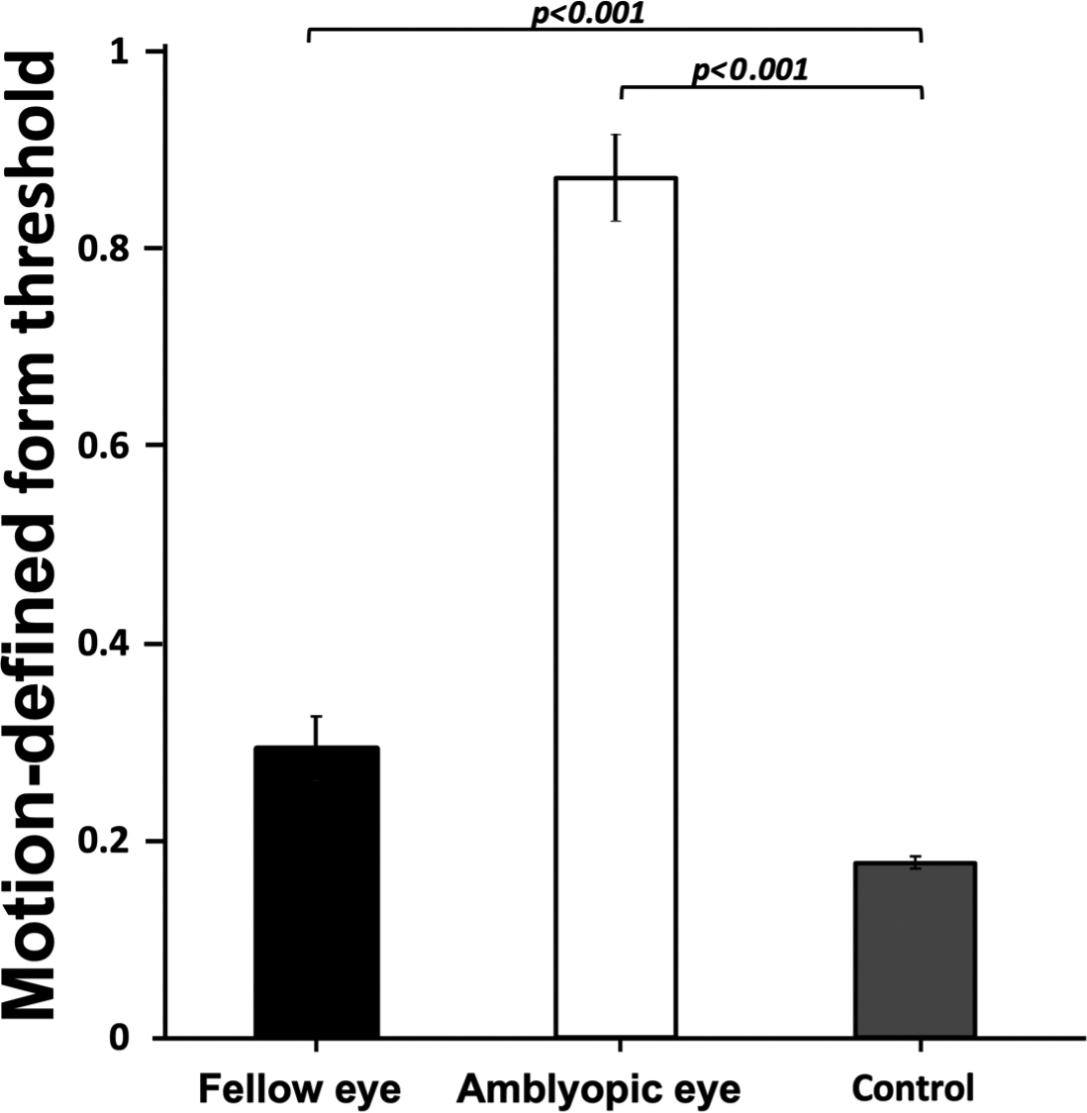
Mean motion-defined form coherence thresholds for control (*N* = 58), amblyopic (*N* = 30), and fellow eyes (*N* = 30). Error bars are standard errors.

Mean (±SD) motion-defined form coherence threshold was 0.87 ± 0.24 when viewing with the eye affected by deprivation amblyopia and 0.29 ± 0.18 for fellow eyes (Fig. 1). Compared with the control group threshold of 0.18 ± 0.05, the amblyopic eye threshold was significantly elevated (t_86_ = 21.1, *P* < 0.001), and the fellow eye threshold was significantly elevated (t_86_ = 4.54, *P* < 0.001). Motion-defined form coherence thresholds were abnormal in 90% of amblyopic eyes and 40% of fellow eyes. All children with abnormal fellow-eye thresholds also had abnormal motion-defined form thresholds in their amblyopic eye.

**Figure 2.**
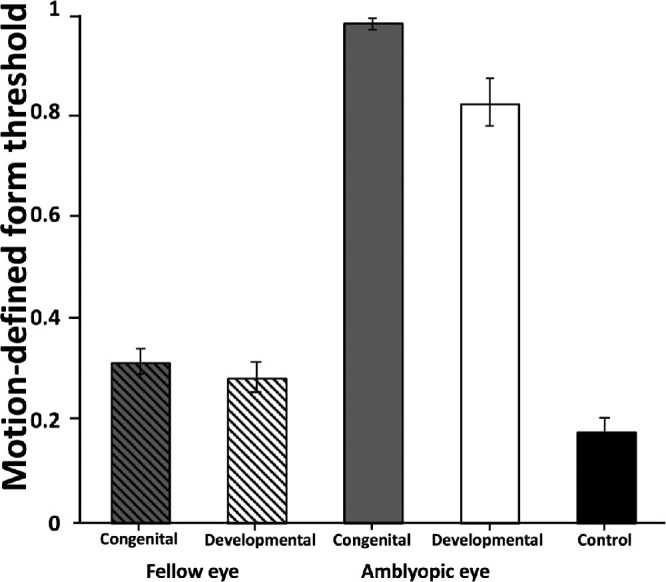
Motion-defined form coherence thresholds in the congenital (*N* = 11), developmental (*N* = 19), and control groups (*N* = 58). Error bars are standard errors.

Motion-defined form thresholds for congenital and developmental deprivation amblyopia subgroups are shown in Figure 2. A 2-way mixed-design ANOVA on the patient z-scores revealed a main effect of eye (amblyopic eye versus the fellow eye; F_(1,28)_ = 140.8, *P* < 0.001) with higher thresholds in the amblyopic eyes, but no main effect of group (congenital versus developmental ) and no group by eye interaction (*P* > 0.05 for both). The percentage of motion-defined form deficits in the congenital and developmental groups was not statistically different in either the amblyopic (100% vs. 84%, 95% confidence interval [CI]_difference_ = −18% to 40%) or the fellow eyes (55% vs. 32%, 95% CI_difference_ = −12% to 52%).

**Figure 3.**
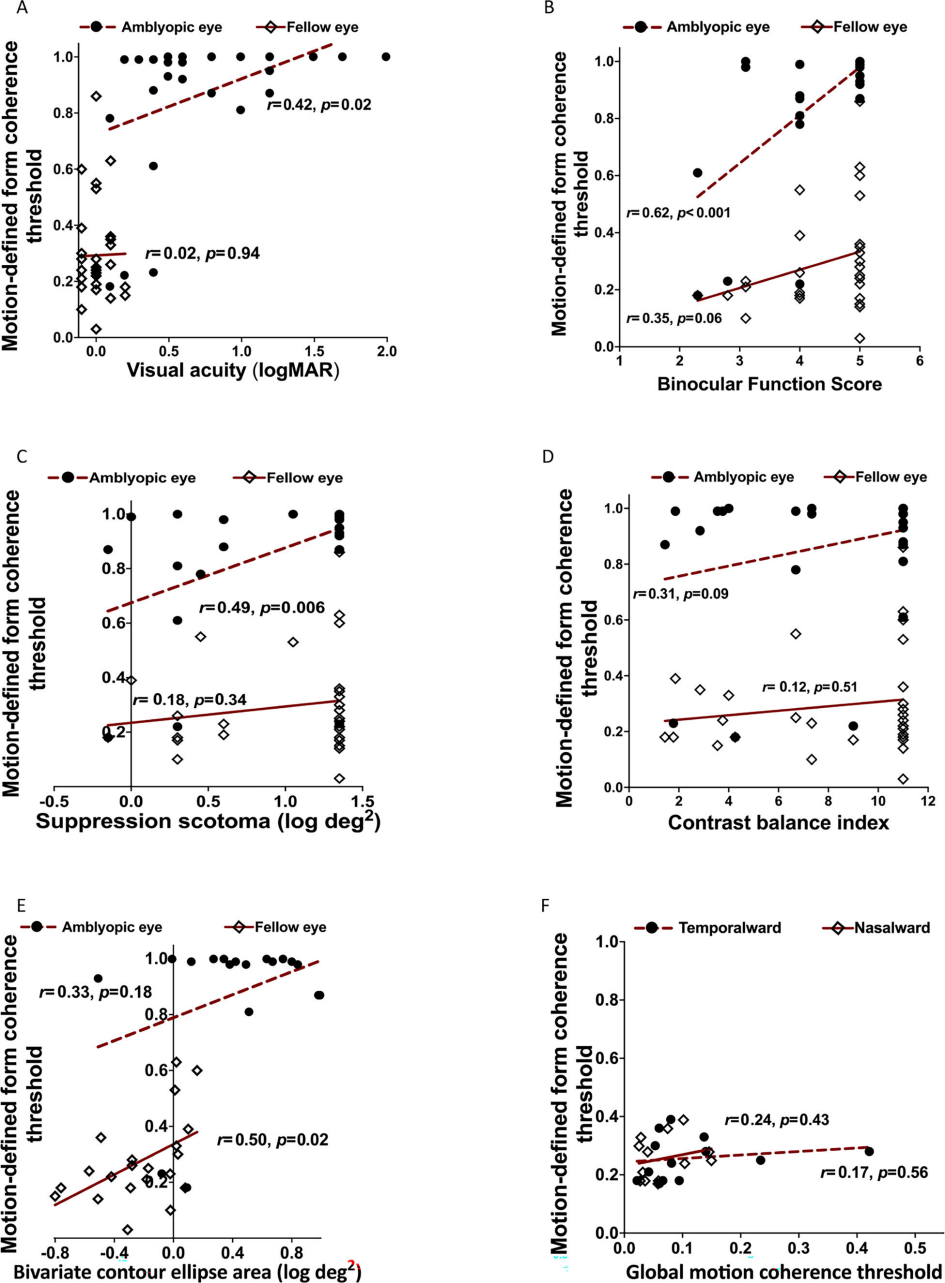
Amblyopia participant correlations between motion-defined form coherence thresholds in each eye and: (**A**) visual acuity in each eye, (**B**) binocular function, (**C**) extent of interocular suppression, (**D**) depth of interocular suppression, (**E**) fixation instability in each eye. (**F**) Correlations between fellow-eye motion-defined form coherence thresholds and fellow-eye nasalward and temporalward global motion thresholds.

There was no significant difference in amblyopic eye or fellow eye motion-defined form thresholds between the children with mild/moderate deprivation amblyopia (0.10 − 0.60 logMAR) and those with severe deprivation amblyopia (0.80 − 2.00 logMAR; amblyopic eyes: t_28_ = 1.95, *P* = 0.06, fellow eyes t_28_ = 0.80, *P* = 0.43). There was a moderate correlation between amblyopic-eye motion-defined form coherence threshold and amblyopic-eye visual acuity (*r* = 0.42, *r*^2^ = 0.18, *P* = 0.02, 95% CI = 0.07 to 0.68; Fig. 3A), and visual acuity accounted for 18% of the variance in motion-defined form coherence thresholds (based on *r*^2^). Fellow-eye coherence thresholds were not associated with fellow-eye visual acuity (*r* = 0.02, *r*^2^ = 0.00, *P* = 0.94, 95% CI = −0.35 to 0.37). This latter finding is not surprising given the small variability in visual acuity, with all fellow eyes less than or equal to 0.2 logMAR (see Fig. 3A).

All 30 children in the deprivation amblyopia group had poor binocular function scores (ranging from 2.3 to 5.0). Binocular function scores and motion-defined form coherence thresholds were strongly correlated for amblyopic eyes, with 38% of the variance in coherence thresholds explained by binocular function score (*r*_s_ = 0.62, *r*^2^ = 0.38, *P* < 0.001, 95% CI = 0.33 to 0.81; Fig. 3B), but not for fellow eyes (*r*_s_ = 0.35, *r*^2^ = 0.12, *P* = 0.06, 95% CI = -0.02 to 0.64; see Fig. 3B).

Amblyopic eye motion-defined form coherence threshold was moderately associated with the size of the suppression scotoma, with 24% of the variance in coherence thresholds explained by this factor (*r*_s_ = 0.49, *r*^2^ = 0.24, *P* = 0.006, 95% CI = 0.15 to 0.73; Fig. 3C), but not with the depth of suppression (CBI; *r*_s_ = 0.31, *r*^2^ = 0.10, *P* = 0.09, 95% CI = −0.07 to 0.61; Fig. 3D), although CBI was elevated in most children. Fellow eye coherence thresholds were not associated with the extent (*r*_s_ = 0.18, *r*^2^ = 0.03, *P* = 0.34, 95% CI = −0.20 to 0.52; see Fig. 3C) or the depth of suppression (*r*_s_ = 0.12, *r*^2^ = 0.01, *P* = 0.51, 95% CI = −0.26 to 0.47; see Fig. 3D).

Unstable fixation (BCEA greater than the upper 95% CI limit for controls of −0.19 log degree^2^)^45^ was observed in 94% of children during monocular viewing with their amblyopic eye, and in 52% with their fellow eye. Motion-defined form coherence threshold was moderately associated with BCEA in the fellow eye, with 25% of the variance in coherence thresholds explained by fixation instability (*r* = 0.50, *r*^2^ = 0.25, *P* = 0.02, 95% CI = 0.08 to 0.77; Fig. 3E), but not in the amblyopic eye (*r* = 0.33, *r*^2^ = 0.10, *P* = 0.18, 95% CI = −0.16 to 0.69; see Fig. 3E). Seventeen (57%) children with deprivation amblyopia had fusion maldevelopment nystagmus, infantile nystagmus, or a combination of both noted in their clinical record and an additional 6 (20%) had mild fusion maldevelopment nystagmus or occasional bursts of fusion maldevelopment nystagmus noted in eye movement recordings, for a total of 23 (77%). Amblyopic eye motion-defined form coherence thresholds were significantly worse in children with nystagmus than those without (0.96 vs. 0.75; t_28_ = 2.65, *P* = 0.01). Fellow eye coherence thresholds were not different between children with nystagmus and those without (0.29 vs. 0.29; t_28_ = 0.00, *P* = 1.0).

Hierarchical multiple regression indicated that binocular function score was the only significant predictor of motion-defined form coherence threshold in the amblyopic eyes; *R*^2^ = 0.40, F(1, 28) = 18.8, *P* < 0.001. None of the other variables (visual acuity, scotoma size, and depth of suppression) were significant predictors, and the amount of variance explained increased by only 4% when they were included in the model; *R*^2^ = 0.44, F(4, 25) = 4.8, *P* = 0.005. There were no significant predictors of fellow-eye motion-defined form thresholds, and the model was not significant; *R*^2^ = 0.11, F(4, 25) = 0.8, *P* = 0.53. A second model using the data for the subset of 18 children with fixation instability data confirmed that only binocular function score was a significant predictor in the amblyopic eyes; R^2^ = 0.42, F(1, 16) = 11.4, *P* = 0.003. In the fellow eyes, BCEA was a significant predictor of motion-defined form threshold; *R*^2^ = 0.35, F(1, 16) = 8.5, *P* = 0.01. None of the other variables were significant predictors and the amount of variance explained increased by only 12% when they were included in the model; *R*^2^ = 0.47, F(5, 12) = 2.1, *P* = 0.13.

Children with deprivation amblyopia had significantly elevated T/N global motion coherence ratios relative to controls (2.00 ± 1.48 vs. 1.08 ± 0.24; t_28_ = 2.51, *P* = 0.01), and 6 of 13 (46%) children with deprivation amblyopia had abnormally high T/N ratios in their fellow eye. Neither temporalward nor nasalward global motion thresholds were correlated with fellow eye motion-defined form coherence thresholds (*P* > 0.05 for all), but the subset of children tested did not include anyone with motion-defined form thresholds above 0.4 (Fig. 3F).

## Discussion

Overall, we found that motion-defined form perception was disrupted in each eye of children with deprivation amblyopia. Larger deficits in amblyopic eyes were associated with poorer binocular function, larger suppression scotomas, and the presence of nystagmus. Only binocular function was a significant predictor of amblyopic-eye coherence thresholds. The larger deficit in motion-defined form perception in amblyopic eyes relative to fellow eyes may be at least partly due to poorer visual acuity, which was associated with higher thresholds, and explained 18% of the variance in amblyopic but not fellow eyes. Motion-defined form perception is known to be influenced by poor visual acuity. For example, we found previously that motion-defined form thresholds in people with typical vision were elevated by blurring lenses once visual acuity decreased to 0.4 logMAR and lower.^46^ Eighty percent of the amblyopic eyes in the current study had visual acuity in this range, but all of the fellow eyes had visual acuity of 0.2 logMAR or better. In addition, motion-defined form deficits were more common in children with deeper (i.e. larger interocular visual acuity difference) strabismic and/or anisometropic amblyopia at the start of occlusion therapy.^43^ However, motion-defined form coherence thresholds were not correlated with amblyopic eye visual acuity in children with treated strabismic and/or anisometropic amblyopia,^16^^,^^21^ and we suggest that assessing form defined by motion contrast in amblyopia provides complementary information to that obtained by standard assessments of visual acuity with optotypes defined by luminance contrast. Future studies with stimulus alterations that take into account the visual acuity of the participant may help to disentangle visual acuity and motion-defined form deficits in amblyopia.

The magnitude and prevalence of disruption in fellow eyes is similar to that reported previously for strabismic, anisometropic, and mixed mechanism amblyopia.^20^ We suggested that fellow-eye motion-defined form deficits in strabismic and anisometropic amblyopia are caused by impaired binocular function rather than by reduced visual acuity.^20^ This conclusion was based on similarities in the prevalence and severity of fellow-eye deficits in children with residual amblyopia and children who recovered normal visual acuity following treatment. It is supported by the failure of monocular occlusion therapy to improve motion-defined form deficits in either eye,^43^ and by lower magnitude and prevalence of fellow eye deficits in children receiving binocular amblyopia treatment.^20^ The mechanism for fellow eye deficits in deprivation amblyopia is less clear. The current study found no association between fellow eye motion-defined form deficits and any measure of binocularity – stereoacuity/fusion, size of suppression scotoma, depth of suppression – although all children had poor stereoacuity and most had elevated interocular suppression (see the Table). We cannot rule out fixation instability as a contributing factor to elevated motion-defined form coherence thresholds in fellow eyes because we found that the former was a significant predictor of the latter, and explained 25% of the variance. This is in contrast to our previous finding in strabismic and anisometropic amblyopia for which fixation stability was not correlated with global motion thresholds.^47^

Cataracts are considered to be congenital when they are visually significant at birth. Cataracts that develop or become visually significant later in infancy or childhood are referred to as infantile or developmental.^48^ Both types of cataract may lead to amblyopia, but the pattern of deficits may differ, and this has been attributed to differences in the sensitive periods across different aspects of vision.^49^ For example, the sensitive period for damage by cataract is long for visual acuity, but relatively short for global motion perception.^30^ We found motion-defined form deficits in each eye with deprivation by congenital or developmental cataracts. These results may not be surprising given that sensitivity to motion-defined form typically matures after the age of 7 years,^50^ and should be at least somewhat susceptible to disruption even by cataracts that developed several years after birth.^51^

Intact global motion perception may be required for good performance on our motion-defined form task.^52^ We assessed this in the fellow eye of a subset of children with deprivation amblyopia by comparing coherence thresholds for temporalward and nasalward global motion. We found a nasalward bias, with higher thresholds for temporalward motion, relative to controls. This motion perception asymmetry has also been found in enucleated individuals,^18^ and is thought to represent immature motion perception.^53^^–^^55^ Consistent with this interpretation, most children with deprivation amblyopia who showed a nasalward bias in global motion perception also showed deficits in motion-defined form perception, although the association between these two measures was not significant. This suggests a general problem with motion perception in some but not all cases. Note, the dot spacing used to create global motion in the current study was not the best for revealing deficits, and may have underestimated the extent of disruption. We previously found larger deficits in anisometropic and strabismic amblyopia for stimuli with smaller dot spacing.^56^^,^^57^

The motion-defined form stimuli used in the current study have been shown to activate the cortical motion area V5/MT+ and lateral occipital cortex in adults without amblyopia,^58^ with significantly less activation in both areas with fellow eye viewing in adults with strabismic or anisometropic amblyopia.^59^ The global motion stimuli typically activate V5/MT+.^58^ Based on the similarities in motion-defined form and global motion perception deficits across etiological subtypes of amblyopia, we predict that deprivation by unilateral cataract during development disrupts binocular processing in V5/MT+ and other motion-sensitive cortical regions. This has clinical implications for the design of new treatments aimed at improving binocular function as well as monocular deficits in visual acuity.

## Supplementary Material

Supplement 1
